# Perceived barriers to physical activity behaviour among patients with diabetes and hypertension in Kosovo: a qualitative study

**DOI:** 10.1186/s12875-022-01866-w

**Published:** 2022-09-30

**Authors:** Ariana Bytyci Katanolli, Nicole Probst-Hensch, Katrina Ann Obas, Jana Gerold, Manfred Zahorka, Naim Jerliu, Qamile Ramadani, Nicu Fota, Sonja Merten

**Affiliations:** 1 Department of Epidemiology and Public Health, Swiss Tropical and Public Health Institute, Allschwil, Switzerland; 2 University of Basel, Basel, Switzerland; 3 Swiss Centre for International Health, Swiss Tropical and Public Health Institute, Allschwil, Switzerland; 4 National Institute of Public Health Kosovo, Prishtina, Kosovo; 5 Medical Faculty, University of Prishtina, Prishtina, Kosovo; 6 Accessible Quality Healthcare Project, Prishtina, Kosovo

**Keywords:** Barriers, Physical activity, Diabetes, Hypertension, Kosovo

## Abstract

**Background:**

In a cohort of primary health care users across Kosovo (KOSCO cohort), high rates and poor control  of diabetes and hypertension were observed. These conditions can be prevented and better controlled by adapting to a healthy lifestyle. Physical activity is an important target, as inactivity and related obesity were very prevalent in the KOSCO cohort. This qualitative study aims to identify individual and structural barriers to physical activity perceived by patients with diabetes and/or hypertension so as to inform health care providers and policy-makers in Kosovo on strategies for promoting physical activity.

**Methods:**

Interviews were conducted from July to October 2020 with 26 public primary health care users from five municipalities of Kosovo (Mitrovica, Vushtrri, Fushe Kosova, Gjakova, and Malisheva). The qualitative study was nested into the KOSCO cohort. KOSCO was implemented in 2019 and recruited consecutive patients visiting the public primary health care centres in these municipalities. Participants of this qualitative sub-study were selected if they had a doctor’s diagnosis of diabetes and/or hypertension. The interview guide consisted of questions related to physical activity barriers these patients are facing, despite having received motivational counselling sessions in primary healthcare centres. Data were analysed using a framework methodology.

**Results:**

Three main themes moderating physical activity behaviour were identified: 1) neighbourhood built environment, 2) health-related problems, and 3) social support. The barriers to physical activity related to the first theme were structural features of the neighbourhoods such as: crowded sidewalks, lack of green spaces, lack of proper lighting in public spaces, as well as dense traffic. In regards to the second theme, the main health reasons for study participants to delay physical activity were related to: physical discomfort as well as stress, worry, and lack of energy. An additional barrier to exercise was lack of social support specifically from friends.

**Conclusion:**

The study identifies structural and individual targets for integrated and inter-sectoral physical activity promotion efforts.

**Supplementary Information:**

The online version contains supplementary material available at 10.1186/s12875-022-01866-w.

## Introduction

Non-communicable Diseases (NCDs) are becoming increasingly more prevalent in low- and middle-income countries (LMICs). The burden of NCDs is different between LMICs and high-income countries (HICs) considering the drivers like promotion of healthier lifestyles as well as providing relevant government policies [1]. A systematic review of NCD policy in LMICs demonstrated that countries are not well prepared to address the NCD epidemic. National policies to reduce NCDs were developed by only a few LMICs, and physical activity was rarely included in these policies [2].

Data from the Global Burden of Disease (GBD) study identified physical inactivity as a risk factor which accounted for approximately 1.3 million deaths (17 deaths per 100,000 inhabitants) in individuals aged 25 years and over [3]. There is a plethora of evidence showing that physical activity is protective for several chronic diseases. People with type 2 diabetes have the greatest benefit, since physical activity reduces the risk of type 2 diabetes by 33–50% [4].

The increasing prevalence of physical inactivity in LMICs [5] has been identified as an important driver of the increase in age-standardized diabetes incidence and disability-adjusted life years (DALYs) in LMICs over the past decade [6]. Physical inactivity is an important risk factor in the aetiology of cardiovascular diseases and diabetes [7], and a target for supporting the control of glycaemia and blood pressure in patients with diabetes and hypertension [8, 9]. In addition, previous research has shown that usually individuals with diabetes undertake less physical activity then individuals without diabetes [10]. Thus, it is essential to understand what barriers to physical activity patients with diabetes and hypertension face.

Some of the reported barriers to physical activity more evident in the cities are related to urbanization, more specifically built environment, overcrowding, sedentary occupation as well as safety issues, which contribute towards a sedentary lifestyle [11]. In addition, individuals with chronic conditions and multi-morbidity have additional barriers due to higher levels of depression, mobility difficulties or pain, or due to limited respiratory function or cardiovascular fitness, among others [12].

In order to inform public health policy and design efficient interventions that aim to increase the levels of physical activity in specific populations in LMICs, it is important to identify country- and context-specific obstacles [13]. Barriers are not restricted to the level of individual behaviour. In recent years, the role of the structural built environment has been a focus in understanding the causes of physical activity behaviour [14]. The built environment consists of designed spaces that support activities, the infrastructure for transportation systems, such as roads, sidewalks, and bike paths as well as urban design [15]. Walkability, green spaces, as well as perceived safety or aesthetics of the residential neighbourhood have all been associated with physical activity levels in different domains (e.g. cycling; leisure walking) [16]. To create an environment that enables people to live healthier lifestyles, the interaction and integration of factors within and across all levels are addressed through individual, interpersonal, community, organizational and governmental levels [17]. Furthermore, another study highlighted the important role of health care professionals in providing social-environmental support for people with type 2 diabetes [18].

In Kosovo, where the Health Information System (HIS) is under development [19], the country needs reliable data for efficient policy-making toward NCD prevention and control. Therefore the objectives of this study are:

- To identify individual and structural barriers to physical activity perceived by patients with diabetes and hypertension in Kosovo.
- To propose strategies for addressing physical activity barriers in patients with diabetes and hypertension in Kosovo.

## Materials and methods

### Study setting

The study was conducted in the primary health care (PHC) centres in five municipalities of Kosovo (Mitrovica, Vushtrri, Fushe Kosova, Gjakova and Malisheva), where motivational counselling sessions on lifestyle behaviours including physical activity promotion are offered. The counselling sessions were introduced through the Accessible Quality Healthcare Project (AQH) which offered several training sessions on motivational interviewing to nurses working in PHC. This approach of delivering counselling sessions by PHC nurses based on motivational interviewing approach is a way of providing one-on-one sessions to patients through empathic listening, eliciting self-motivating statements, and responding to resistance [20]. Motivational counselling based on motivational interviewing techniques for health behaviour change was shown to be effective in physical activity self-management in people with chronic conditions [21] such as type 2 diabetes [22] and hypertension [23].

AQH is a project of the Swiss Agency for Development and Cooperation (SDC), implemented by the Swiss Tropical and Public Health Institute (Swiss TPH). Its aim is to work with local stakeholders to improve the quality of PHC in the public health sector, with a focus on the prevention of NCDs, and in particular diabetes and hypertension. The reason for focusing on those two conditions is that motivational counselling is delivered to tackle physical inactivity, smoking, unhealthy diet and alcohol consumption which are common risk factors for diabetes and hypertension. To evaluate the performance of certain areas of the AQH project, as for example the counselling sessions, the Kosovo Non-Communicable Disease Cohort (KOSCO) was designed. KOSCO was initiated in 2019 by recruiting consecutive PHC patients aged 40 years or older for interview and health assessment, as described elsewhere [24].

### Study design

With an embedded design, a qualitative descriptive study was nested in the KOSCO study, applying a framework approach to data analysis.

### Study population

The participants were identified through purposive sampling. All patients who received at least one motivational counseling session and who had a diagnosis of diabetes and/or hypertension were considered eligible for this qualitative study. In all 5 Municipalities, we selected the first eligible participant from the KOSCO database. We then selected the next person with matching the criteria and chronologically advanced through the database list. Interviews started simultaneously in all 5 Municipalities. Initially, we intended to do 30 interviews but after the 26th interview, we decided to stop as no new themes were emerging.

### Data collection

In-depth interviews were conducted with 26 KOSCO participants between July and October 2020. The recruitment of study participants was stopped once data saturation [25] was achieved. A semi-structured interview guide was developed in English and translated into the Albanian language for pilot-testing and adaptation. The interview guide consisted of questions related to specific barriers to physical activity behaviour that patients may face after having received motivational counselling sessions. The questions also focused on healthy nutrition, non-smoking, obesity prevention, and physical inactivity (Supplementary file 1: Interview Guide). The first author (ABK), a female researcher with a background in public health, conducted the interviews in the Albanian language. ABK was referred to a published guide “Interviewing as qualitative research” [26] to conduct the interviews based on scientific methodology. Due to the COVID-19 pandemic, telephone instead of in-person interviews were conducted in order to avoid participants’ risk of potential infection. The interviews lasted between 30 and 40 min, were audio-recorded through Open Data Kit (ODK) software and uploaded to a secure server at Swiss TPH. The interviews were transcribed verbatim in Albanian and the first ten transcripts were translated into the English language to share with the senior author (SM).

### Data analysis

Framework analysis as outlined by Gale et al. [27] was used to extract themes and sub-themes from verbatim-transcribed interviews. Data collection and analysis took place iteratively. Initial open coding was conducted by the first author fluent in Albanian. The senior author read the 10 translated interviews and reviewed the initial coding, and together the first author and senior author agreed on a working analytical framework after coding the first ten transcripts and grouping the codes into categories. The remaining transcripts were indexed by applying and expanding the initially developed codes and categories; additional codes evolved after a detailed re-reading of all transcripts. This way, themes related to perceived barriers toward physical activity in patients with diabetes and hypertension were retrieved. Previous research has shown that the analysis of qualitative data could be used as an effective way to inform policy decision-making by taking into account patient experiences [28]. The involvement of patients can also guide healthcare priorities, which may improve community health [29].

### Ethical considerations

Ethical approvals for the study were obtained from Ethics Committee Northwest and Central Switzerland (Ref. 2018–00994) and the Kosovo Doctors Chamber (Ref. 11/2019). Prior to data collection, verbal informed consent was obtained before the phone interviews, whereas after COVID-19 lockdown measures were released, written informed consent forms were obtained from all the participants that were interviewed.

## Results

### Description of study participants

Table 1 shows socio-demographic characteristics of the interviewed participants. Participants were mainly from urban areas, and there was a similar distribution of men and women. The mean age was 57 and the majority were married. In regards to education, the majority have completed high school, and there was no significant difference in the educational level between men and women. Concerning employment, out of 26 participants, only 9 were employed, 4 participants were in retirement age (65 years or older), and 13 participants were not employed. Eleven study participants self-assessed to have a moderate socioeconomic status (SES), 8 reported to have good SES, and 7 stated to have poor SES.

**Table 1:** Socio-demographic characteristics of interviewed participants by municipality (*n* = 26)

Socio-demographic characteristics	
**Municipality**	
Mitrovica	5
Vushtrri	6
Fushe Kosova	5
Gjakova	5
Malisheva	5
**Residence**	
Urban	16
Rural	10
**Age [years]**	
Mean (SD)	57 (5.9)
Range	41–66
**Sex**	
Men	14
Women	12
**Marital status**	
Married	24
Widowed	2
**Education**	
Primary school	9
High school	16
University degree	1
**Employment status**	
Not in employment	13
Retirement age	4
Employed	9
**Socio-economic status (SES)**	
Good	8
Moderate	11
Poor	7

Table 2 summarizes the sub-themes identified in each main theme. Qualitative findings are presented based on emerging themes and sub-themes from in-depth interviews with study participants. The main themes related to barriers for physical activity were: neighbourhood built environment, health-related problems, and social support.

**Table 2:** Main themes and sub-themes arising from transcript analysis

Main themes	Sub-themes
Neighbourhood built environment	Neighbourhood infrastructure
	Lack of green spaces
	Dense traffic
Health-related problems	Physical discomfort and pain
	Lack of energy, stress, and worry
Social support	Lack ofpport from friends

### Theme 1: Neighbourhood built environment

Study participants expressed that neighbourhood built environment was one of the main reasons which limited them to be physically active. Issues were related to the road infrastructure, lack of green spaces, and dense traffic in the neighbourhoods.

#### a) Neighbourhood infrastructure

Since study participants were affected by diabetes and/or hypertension, the first choice of physical exercise reported by these study participants was walking in their immediate neighbourhood. Most participants described various barriers as pedestrians they were facing when they went walking. Crowded sidewalks in the neighbourhoods was one of the main concerns perceived by study participants that prevented them to be engaged in exercise such as walking.

*“Well it’s a problem, you don’t have places where to walk since there are no free sidewalks and because there are lots of cars that park on sidewalks and they are usually not free” (55 years old, male, Mitrovica).*

Mainly, the sidewalks were either crowded with cars or they were not properly maintained:

*“When I want to go to walk in my neighbourhood it’s not good because on some roads the sidewalks are cracked … and you can’t walk freely, that’s why I have to go somewhere far in the mountains during the day, and I need more time for that” (59 years old, male, Malisheva).*

The lack of proper street lighting was another issue for individuals that wanted to walk after working hours, which required them to go further away to exercise:

*“In the area where I live we don’t have proper lights, and I can’t go after work to walk. They also said that they will make a walking trail but still, they didn’t do it. But during weekends I try to go with the family somewhere further away from where we live and we can all walk around and play soccer” (55 years old, male, Mitrovica).*

These respondents also mentioned the inconvenience of having to go further away from home to exercise due to poorly maintained infrastructure. Such a thing required more time and effort, and physical activity was likely constrained to the weekends. To be engaged in physical activity during the week was especially challenging for individuals who work since they would need to make time when there is still daylight outside.

### b) Lack of green spaces

Another identified barrier was the lack of green spaces close to the neighbourhoods; participants have to walk a long time until they would arrive at the parks/green areas or they would need to take their cars to go for a walk or do exercise.

*“ ….. there is a problem to go out and walk in the neighbourhoods as we don’t have a park close by. We usually go away from where we live to find more quiet and green areas, which is far away and we can’t walk there, so we have to take our car” (61 year old, male, Vushtrri).*

The lack of green spaces and parks close to neighbourhoods was also highlighted by participants with families who would like to have playgrounds for children and at the same time areas for playing or walking together.

*“Where I live in my neighbourhood there is no park or place for children to play. We need somewhere nice to go out with our families to play or to walk together. I am with diabetes and it is very difficult for me to do what my doctor said, like to go to walk, it is not easy when there is no park close to where you live” (50 year old, female, Malisheva).*

#### c) Dense traffic

In addition to constrained space for pedestrians, dense traffic further inhibited physical activity in people’s neighbourhoods. First, due to high traffic flow, walking was not safe in some neighbourhoods. Second, the overcrowded sidewalks with parked cars obstructed these patients from walking freely in their communities.

*“Well I have a place to walk here in Shupkovc, it’s half an hour away and there are no cars there. But in my neighbourhood where I live it’s dangerous to walk, they drive very bad and people need to be really careful, also they park everywhere and there is no free space for us who want to walk” (54 year old, male, Mitrovica).*

### Theme 2: health-related problems

The second theme that emerged was related to health problems due to diabetes and hypertension. Most of the participants reported that one of the main reasons why they did not exercise was that they had some sort of health-related issue. Two sub-themes were identified: physical discomfort and pain; as well as lack of energy, stress and worry.

#### a) Physical discomfort and pain

Physical discomfort was reported to cause difficulties to begin and do any kind of exercise, such as numbed feet and back problems.

*“I sometimes have problems with my feet and they hurt me, and sometimes can’t go to walk because of that”* (59 year old, male, Malisheve).

Some participants did not even walk or do any other type of exercise because they were afraid that exercise would aggravate their health problems.

*“I walk slowly, but to do exercises I can’t because I have a problem with the heart, I have diabetes and also my back hurts, so I just do something light. Sometimes my feet get numb and because of these health problems I can’t walk or do any exercise*” (54 year old, female, Vushtrri).

Furthermore, an important point outlined by study participants was that they were not knowledgeable about the types of exercises they could perform due to their health conditions. One participant stated that they are concerned and expressed fear that they could hurt themselves if they would exercise without somebody else’s help.

*“I have a lot of back pain, and try to exercise at home but I only do it sometimes, I don’t know if I am allowed to do exercises alone without somebody telling me what to do….I am scared that my back will hurt more”* (64 year old, female, Gjakova).

A recurring problem for several participants seemed to be not knowing what type of exercises they could do without compromising their particular health condition or triggering more pain.

#### b) Lack of energy, stress and worry

Several participants outlined the problems related to lack of energy, stress and other worries, which prevented them from making the move to exercise. Some participants mentioned that even going outside for a walk was not enjoyable for them, and preferred to stay inside their houses.

*“I am very stressed and I can’t go to walk and do anything, I don’t like it, I just stay inside”* (52 year old, male, Malisheve).

Low levels of energy were specifically demotivating in regards to physical exercise.

*“Sometimes I have low energy and don’t feel like going to do exercise. Some days I have no energy at all and some days it gets better”* (59 year old, female, Fushe Kosova).

Another participant mentioned worries related to family issues which influence the decision to start exercising. The aspects of stress, worry, hopelessness, and lack of energy could be linked to mental health problems, which these participants might have but are not aware of.

*“When I go out to walk I get tired very fast, I don’t have a lot of energy and I don’t enjoy it as much because I think about all the things. For example, my son is not working, my daughter has finished only her high school, we did not have the money to send her to finish her university, and I think about all of these things”* (65 year old, female, Gjakova).

### Theme 3: social support

The study findings show that social support has a role in motivating the study participants to exercise. The main support mentioned by participants was the importance of their peers to do activities together and how there is a lack of engagement with them for physical activity.

### Lack of social support from friends

Some participants expressed that lack of continuous support and contact with friends to engage in physical activity was another barrier to physical activity. It was stated that participants would mainly go out with friends to socialize or to drink coffee, but not to exercise.

*“My friends can’t help me for exercising. When we meet we only go to drink coffee and get together for socializing. Probably to go together with other people I would go and exercise more”* (54 year old, male, Mitrovice).

Study participants reflected that changes they do specifically for physical activity behaviours are initiated by themselves and/or get help from family members, but friends were not part of it. Also, participants noted that they would prefer to exercise with a friend since it could motivate them.

*“Friends do not have time to help me, sometimes my family does help me, but it would be nice to go and walk with a friend or to go and exercise together with a group. By myself I can’t go”* (56 year old, female, Mitrovice).

Furthermore, it was outlined that some participants do not have social interactions and would actually prefer to go walking with other people.

*“I don’t really have that many friends. I mainly stay by myself, maybe if there would be someone else to go out with I can go out and walk together with them. It is better when there are more people you do everything easier”* (64 year old, female, Gjakova).

## Discussion

This study investigated the perceived barriers to physical activity behaviour in patients with diabetes and hypertension. Even though these patients had received one-on-one motivational counselling sessions, several structural and individual barriers prevent these patients from being physically active.

The first derived theme from our study is related to the neighbourhood built environment, which encompasses structural barriers to physical activity. Many structural aspects in the neighbourhoods, such as lack of proper lighting, crowded sidewalks, lack of green spaces, and dense traffic were mentioned by the study participants. This is in line with previous research, which equally highlighted the negative effect of poor infrastructure, such as irregular roads and pavements [30], neighbourhood insecurity [31], or the lack of recreational facilities on physical activity of patients with type 2 diabetes [32]. A higher neighbourhood walkability supports physical activity also in overweight and obese adults with metabolic syndrome [33]. Global review and meta-analysis further found that neighbourhood walkability and access to green space is associated with a reduced risk/prevalence of type 2 diabetes, thus also playing a key role in primary prevention of this disease [34]. A qualitative systematic review equally confirmed the importance of access to recreational facilities, green open spaces, and rest areas as the most relevant environmental factors enabling older adults to be physically active [35].

The second emerging theme of perceived barriers to physical activity in our study was health-related problems, which are individual barriers to physical activity. Study participants reported that the main health-related reasons for not exercising were physical discomfort, pain, stress as well as lack of energy, and other worries related to mental health problems. Our results confirm findings from an earlier study that identified pain, poor health, depression, and tiredness as common internal barriers to exercise among persons with type 2 diabetes or persons at high risk for diabetes [36]. Fatigue is also considered as a barrier for diabetes self-management [37] which influences levels of physical activity in these patients. Pain and physical discomfort as outlined by our study participants were additional individually perceived barriers to physical activity. These findings are consistent with the literature on barriers to physical activity in adults with type 2 diabetes mellitus such as muscle and joint pain [38, 39], physical discomfort and tiredness, [40] and fear of injury [41]. Likewise, in people with obesity, barriers to physical activity that were established in previous studies included pain or physical discomfort, low capability of self-management, lack of time, and fear of injuries [42, 43]. For the special group of adults with morbid obesity, a qualitative systematic review on experiences with physical activity interventions highlighted the importance of addressing experiences of suffering and well-being, the capability of doing physical exercise, and the dimension of belonging with others, when intervening. The experiences of suffering or well-being during physical activity affected the identity of these individuals, which could be either positive or negative; furthermore the authors point to the relevance of a language of dignity when promoting change [44]. Another qualitative meta-ethnography which looked at ageing as a life phase with its own challenges, equally assigned physical activity an important role in helping to regain feelings of purpose and self-esteem, and having a positive effect on identity [45]. Physical inactivity has an effect in the development of comorbidities and is associated with increased mortality [46]. Our study participants reported that health-related problems, as well as neighborhood infrastructure such as lighting, cracked and congested pavements, were barriers to physical activity. Considering the present study’s results, it would be reasonable to expect that walking on pavements that are not well maintained or easily accessible might be even more difficult for people experiencing health problems such as pain.

The third theme focused on the importance of social support; as our study participants reported a lack of social support specifically from friends as an important individual barrier to physical activity. Several study participants outlined that during the times they exercised, they would do it by themselves, but if they would go with a friend, they would probably exercise more. Previous research already pointed to the importance of social support for sustainable behaviour change. Among middle-aged and older adults, social influences are important facilitators of physical activity [47]. It was shown that if middle-aged and older adults exercised alone, physical activity was discontinued over time; therefore, exercising alone is an independent risk factor for physical inactivity [48]. Low social support was also associated with low physical activity among patients with type 2 diabetes [49], and social support from friends was less common compared to that from family members [50]. In the same line, our study participants mentioned that they had support from family to initiate their physical activity behaviour but there was a lack of support from friends. Considering that family ties are strong in the Kosovo social environment, it is expected to have strong family support, but this may not replace a strong social network of friends. Indeed, the importance of social support cannot be overstated. Low social support also impacts the risk of chronic disease through pathways other than physical activity. For example, low social support was an important factor in the development of type 2 diabetes: participants who perceive themselves as having little social support had significantly poorer blood glucose control in stressful situations [51].

### Proposed strategies to address physical activity barriers

**Fig. 1:**
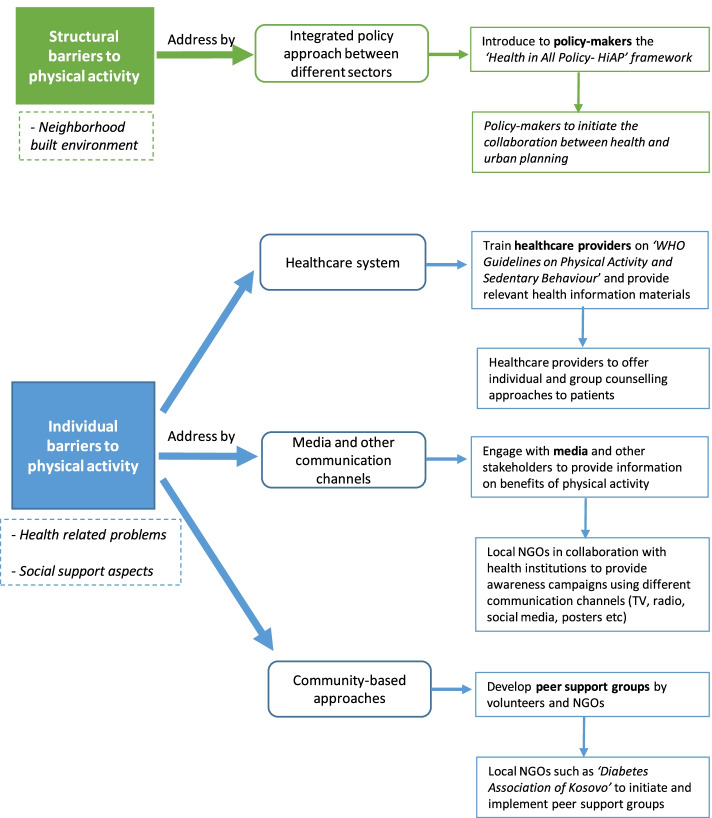
Proposed strategies to address physical activity barriers for patients with diabetes and hypertension

Based on scientific literature and referring to the study findings, we report and explain trans-sectoral strategies that might be found useful to address structural and individual barriers in order to improve physical activity among patients with diabetes and hypertension in Kosovo. An integrated policy approach between different sectors in line with the Health in All Policies (HiAP) framework by the World Health Organization [52] could best address structural barriers such as the neighbourhood built environment. As a complement, individual and group counselling approaches through the healthcare system could best tackle individual barriers such as health-related problems. In addition, media and other communication channels, as well as community-based approaches play a role in addressing social support aspects. Figure 1 illustrates the ways how perceived barriers to physical activity among these patients could be addressed.

#### a) Implications for urban planning

As outlined in the HiAP framework, physical activity behavior for communities could be facilitated by using a collaborative trans-sectoral approach. The HiAP framework takes into account the health implications of public policies and decisions in order to avoid negative health impacts [53]. In the context of urban policies to promote public health interventions, considering the HiAP framework is key to local decision-making processes [54]. Additionally, it must be considered how the already built environment plays a role in modifying or worsening health inequities [55].

The coordination between health and urban planning was shown to be important in improving the health of populations through the Healthy Urban Planning Initiative, where the importance of urban planning for health was recognized by WHO [56]. The design of urban environments is likely to contribute significantly to physical activity in general [57] and for patients with type 2 diabetes in particular [34]. Interventions that were designed to influence active transport equally showed a high impact on physical activity [58]. Yet, more evidence is still needed specifically on the influence of urban space on physical activity in specific patient groups.

#### b) Implications for healthcare providers

Study participants described health-related problems as an important aspect that hindered their physical activity behaviour. This is in line with previous research highlighting the importance of physical co-morbidities and related cognitive problems, sleep/energy problems, or depression, which are all known barriers to physical activity [12]. Our findings further found that lack of energy was a barrier to physical activity for patients with diabetes and hypertension. Furthermore, study participants expressed fears that they could injure themselves since they did not know what kind of exercise they could do in view of their health condition.

First, in order to improve the practice of physical activity among patients with diabetes and hypertension, it is important for healthcare providers to offer tailored information on types of exercises that these patients can perform regardless of their health condition, and to provide advice on how best to overcome low energy levels. By receiving respective training, healthcare providers will have greater confidence in discussing physical activity and exercise with patients, as well as give tailored information to patients [59]. According to ‘WHO Guidelines on Physical Activity and Sedentary Behaviour’ for adults living with chronic conditions, physical activity can be undertaken as part of recreation and leisure (games, play, sports, or planned exercise), transportation (walking, cycling), as well as work or household chores [60]. Second, it is suggested for healthcare providers to offer counselling on physical activity that patients with chronic diseases are encouraged to do according to WHO guidelines. In order to reach as many healthcare providers as possible and train them to be competent in offering counselling on physical activity, an online platform can be used to improve their knowledge. The Cambridge Diabetes Education Program, an online platform tested among healthcare providers in Australia, showed that the addition of online education increased the training uptake among nursing staff [61]. An additional barrier which could be addressed by healthcare providers is the lack of social support for involvement in physical activity. Healthcare providers within PHC centres can organize group physical activity sessions and set goals for doing physical activity together with their social circle. Reduced risk of cardiovascular events was observed when healthcare providers enhanced positive social support by encouraging patients to set goals with family members to do physical activity at a greater level [62]. In medical practice, physical activity counselling and support for the prevention and treatment of chronic diseases should be delivered by the health care systems [63], where healthcare providers at PHC level have a crucial role in addressing individual perceived barriers for physical activity, which are patients’ health-related problems and aspects of social support.

#### c) Implications for local NGOs

Based on WHO Global Action Plan on Physical Activity 2018–2030, it has been proposed to implement sustained public education, and awareness campaigns using traditional, social, and digital mass reach communication channels, combined with community initiatives [64]. In the Kosovo context, to reach patients with diabetes and hypertension through media, it is recommended for local Non-governmental Organizations (NGOs) in collaboration with health institutions to provide awareness campaigns using different communication channels (such as TVs, radio, social media etc).

Support is characterised by receiving empathy which in turn increases the well-being of the patients. Furthermore, an approach that shares practical aspects of managing diabetes in day-to-day life i.e. the ‘how to do’ rather than the ‘what to do’ is preferable. Peer support can involve individual or group approaches with face-to-face, telephone, and internet contacts [65]. Therefore, we propose community-based approaches to address the social support aspects as outlined by the study participants. The peer support groups can be developed and implemented through local NGOs, such as “Diabetes Association of Kosovo’. Past literature has demonstrated that reciprocal peer support holds promise as a method for diabetes care management [66].

## Conclusion

In conclusion, physical activity promotion in people living with diabetes and hypertension can be achieved by addressing individual and structural barriers through integration of multiple sectors such as the health care system, urban planning, communication, and community-based approaches.

### Strengths and limitations

One of the main strengths that this study provides is insights on barriers to physical activity that patients with diabetes and hypertension in Kosovo are faced with daily. Furthermore, this study proposes ways on how these barriers could be tackled through different strategies, more specifically through policy-making as well as the healthcare system.

One of the study’s limitations is that in-depth interviews were conducted by telephone due to the coronavirus pandemic. During telephone interviews, non-verbal cues and body language from study participants could not be observed. On the other hand, evidence shows that telephone interviews are a good medium for data collection [67]. Another limitation is that in this study we only present the barriers to physical activity of the patients that received motivational counselling sessions. Therefore, we did not capture the barriers to physical activity of people that did not receive the intervention. To have a broader overview of barriers to physical activity among patients with diabetes and hypertension, more research is needed on participants that do not seek motivational counselling sessions at PHCs. The participants were recruited from a small number of health facilities. Therefore, further research in other health facilities in the country is needed to complement the barriers to physical activity among people living with diabetes and hypertension identified in this study.

## Supplementary Information

**Additional file 1.**

## Data Availability

The datasets used and analysed in this study are available upon reasonable request by e-mailing the corresponding author.

## Abbreviations

AQH: Accessible Quality Healthcare
HiAP: Health in All Policy
HIS: Health Information System
KOSCO: Kosovo Non-Communicable Disease Cohort
LMIC: Low Middle Income Country
NCDs: Non-communicable Diseases
ODK: Open Data Kit
PHC: Primary Health Care
SDC: Swiss Agency for Development and Cooperation
SES: Socio-economic status
Swiss TPH: Swiss Tropical and Public Health Institute
WHO: World Health Organization
