# Supplementary material for: Perceived barriers to physical activity behaviour among patients with diabetes and hypertension in Kosovo: a qualitative study
Source: BMC Prim Care. 2022 Sep 30;23:257. doi: 10.1186/s12875-022-01866-w (PMC9523175; doi:10.1186/s12875-022-01866-w)
Supplement: Supplementary file 1 — Additional file 1. [file 12875_2022_1866_MOESM1_ESM.pdf]

Interview Guide
Qualitative study

Thank you again for your participation in the study, I will start by asking some questions regarding your experience in the Health Resource Center…

1) How did you hear about the Health Resource Center within the Main Family Medical Center?

    a. PROBE: Did your family doctor refer you to the Health Resource Center to receive counselling from the nurse?
    b. PROBE: Did the doctor explain to you why you were going to see the nurse? If yes, then do you remember what the doctor said?

2) What services did you receive in the Health Resource Center?

3) How many one-on-one sessions did you have with the nurse within the last year?

4) Could you please **describe** in detail how one of the sessions you received from the nurse at the health resource center looked like?
    a. PROBE: When you think about the way the nurse talked to you, how did you feel about it? (Probe: how easy was it to follow; how well could you understand what the nurse said; how did you feel about the tone; did you feel respected; …..)
    b. PROBE: Could you say more about your relationship with the two nurses at the Health Resource Center over the course of the sessions?

5) Were there things about your lifestyle that you wanted to change for your health? If so, what were they?

6) Did you start changing your lifestyle /habits (physical activity, nutrition, smoking, alcohol)? If yes, which ones? How long? How has it been going for you?

7) Which habits (physical activity, nutrition, smoking, alcohol) did you **change**/ **start to change**?
    a. PROBE: **How long** has it been since you changed/started to change your behavior/s?

8) Since having visited the Health Resource Center, how would you consider your health? Has it changed? Why or why not?
    a. PROBE: If so, how did others around you react to your changed way of living?

9) Have the **sessions helped** you to start thinking to change your habit/s (physical activity, nutrition, smoking, alcohol)? If no, why not?
    a. PROBE: How have these sessions made you start thinking to change your habit/s?
    b. PROBE: What exactly motivated you to wanting to change something?

Interview Guide

10) Were you able **to start** changing your physical activity habits/smoking habits/nutrition habits/alcohol habits (we would ask the habit/s they mentioned in Q.7)? If so, how?
    a. PROBE: How did you start to change your specific habit/s? (Please describe the things you did to start changing your habit/s)
    b. PROBE: How well did it go to start changing your practices?
    c. PROBE: How do you **motivate** yourself?- Could you give me an example

11) Did you encounter any **problems or obstacles** when you **started** changing your specific health behavior/s (quitting smoking/eating healthy/exercising/reducing alcohol consumption)? **If so, what were they?**
    a. PROBE: What specific factors and things in the *local community/social networks/personal* were making it **difficult** for you to start to change your habit/s?
    b. PROBE: What were some of the **challenges** that you were facing when you **started** to change your habit/s?

12) How do you feel about the **difficulties** you experienced when you tried to **start to change/maintain** your behavior?
    a. PROBE: What did you do when you wanted to **start to change/maintain** your behavior and for some reason you failed? What type of **help** did you reach out for?

13) Were you changing your habit/s **alone** or with the **help of a family/friend**?
    a. PROBE: How helpful and **supportive** were your family and friends when you were starting to change your habit/s?
    b. PROBE: How did your family and friends **view** your new way of living?

14) Did anything **help** you to **start** changing your specific habit/s (quitting smoking/eating healthy/exercising/reducing alcohol consumption)? If so, what helped?
    a. PROBE: What specific factors and things in the *local community/social networks/personal* **helped** you to start live healthily?

15) Could you tell me what specific **needs** you might have which would help you to change your specific health behavior/s? -Could you give me examples
    a. PROBE: What would help you be more active, eat healthier, quit smoking, reduce alcohol consumption?

16) Can you talk about your new habits/lifestyle? Are you able to maintain it? Why or why not? What works for you personally to live healthily?
    a. PROBE: What **helped** you maintain those changes?
    b. PROBE: What **problems** do you encounter while maintaining your habit/s? (for *personal, social network, community level)*
    c. PROBE: What are some of the **challenges** that you are facing while **maintaining** your specific habit/s? - Can you elaborate more on this?

17) Did your **habits** (nutrition/exercise/smoking/alcohol) /**practices** change over time? If so, how?

18) Between men and women, do you see any differences in terms of health and lifestyle? If so, what?
    a. PROBE: Do you think it is easier for **men or women** to live healthier and change their habits? Why?

20) Overall, what did you **think of the one-on-one sessions**? In general, were you satisfied with the one-on-one sessions delivered by the nurse?

    a. PROBE: What did you **like most** from the one-on-one sessions?/ What aspects of the sessions have made you satisfied most?
    b. PROBE: What do you **wish to change/improve** from the one-on-one sessions?

19) Have you been able to **discuss** with the **nurse the difficulties**?
    a. PROBE: How have the nurses helped you overcome the failures you were experiencing during this process?

20) How did the **nurse help** you **change/maintain** your specific habits?
    a. PROBE: Could you explain this further?

21) Did you read any of the education materials (flyers) that you received?
    a. PROBE: If yes, did the flyers have an impact while you were changing your habits?
    b. PROBE: What else would you have liked to help you change your habits?

22) What other **services or activities** in the **Primary Healthcare Centers** do you think are needed to help you change your specific habit/s?

23) What recommendations do you have for policy-makers in your Municipality to make your surroundings (**local community**) 'healthier' so it would be easier for you to modify/maintain your health behaviors (physical activity, nutrition, smoking, alcohol)?

24) Is there anything else you would like to add?
